# A Case Report of Hirschsprung’s Disease in a Neonate: Early Detection and Review of Management

**DOI:** 10.7759/cureus.33680

**Published:** 2023-01-12

**Authors:** Fatimah Rajabally, Rama Alkhaldi, Helen Huang, Mohammad Said, Farah Elnakoury, Chaithanya Avanthika, Fouad Abdool

**Affiliations:** 1 School of Medicine, Royal College of Surgeons in Ireland, Dublin, IRL; 2 Medicine and Surgery, Karnataka Institute of Medical Sciences, Hubli, IND; 3 Pediatrics, Wellkin Hospital, Moka, MUS

**Keywords:** frozen suction rectal biopsy, review of management, case report, duhamel, hirschsprung's disease

## Abstract

Hirschsprung’s disease is a rare disease characterized by the complete absence of ganglionic cells in the colon, thereby causing loss of peristalsis movement of the bowel. Most cases are diagnosed before the age of one. Here, we present a case of a newborn baby boy who was not feeding well and then developed a distended abdomen and began bilious vomiting. Blood mucoid stools were also observed. The diagnosis of Hirschsprung’s disease was confirmed through a full-thickness rectal biopsy, and the Duhamel surgical procedure was performed as a course of treatment all within the first few days of birth. No complications were reported, and the baby was safely discharged after seven days. This case demonstrates the importance of timely treatment after prompt diagnosis due to the early recognition of the severe symptoms. Even though this disease is rare, pediatricians should be trained to recognize and treat the child to prevent further detrimental outcomes.

## Introduction

Hirschsprung’s disease (HSCR) is distinguished by the absence of ganglionic cells in the submucosal and myenteric plexus contained in the intestines [1]. Aganglionosis commences in the distal rectum and can affect the entire gastrointestinal tract [1,2]. Despite etiopathogenic pathways not being very clear, the progress made in recent times has been significant [2]. Notwithstanding partial knowledge about the etiopathogenic mechanisms, studies have unveiled parts of the embryonic development [2]. The most distinctive factor involved in the etiopathology of this disease is the neural crest disorder, causing the absence of these neural cells [3]. The nervous system belonging to the intestine is derived from the neural crests, specifically the vagal area [2,3]. These cells migrate and occupy the intestine as multipotent progenitor cells and then differentiate in the glial cells, which is yet to be researched and investigated [1,3,4].

HSCR has been proven to be a hereditary condition with 10%-20% of cases with a positive family history. The condition occurs in both males and females; however, studies have shown that it is three times more prevalent in males than in females [3,5,6]. The congenital etiology of HSCR is multifactorial and is prevalent in 1:5000 live births [3]. Most HSCR cases are diagnosed before the child reaches one year of age [7].

HSCR can be classified into three types: long segment, short segment, and total colon aganglionosis [4,8]. In around 80% of patients, short-segment HSCR often affects the rectosigmoid region of the colon and remains the most common form after long-segment HSCR (proximal sigmoid colon, 15%-20%) and colonic aganglionosis (entire colon, 5%) [9].

Symptoms that arise include HSCR-associated enterocolitis, intestinal obstruction, intestinal perforation, and gastroenteritis [6,10]. Surgical removal of the aganglionic segment of the intestine is the inevitable approach, even though it has been recorded that 30%-50% of patients experience symptoms post-surgery [6]. However, advancements in pediatric surgery are directly correlated with the improvement of short-term outcomes regarding HSCR surgical management [6,11].

Consequently, with advancements in surgical treatment for HSCR, mortality has significantly decreased and ranges between 1% and 10% [12]. Nevertheless, up to one-third of patients can develop HSCR-associated enterocolitis, a life-threatening complication that significantly contributes to mortality [10].

In this article, we present a rare case of HSCR in a newborn who was diagnosed and treated successfully all within the first few days of birth, preventing serious complications.

## Case presentation

A 26-year-old female (primigravida) underwent an emergency cesarean section (C-section) at 40 weeks after failed induction. The surgery was completed under spinal anesthesia, and a male neonate was delivered (birthweight: 3.47 kg, length: 51 cm). There was no history of complications during pregnancy, and all the prenatal sonographic exams were deemed to be normal. The newborn examination was normal with no dysmorphism noted. There were no issues following the C-section, and the baby was transferred to the nursery for routine care and feeding.

However, 24 hours post-delivery, the baby was moved to the neonatal intensive care unit (NICU) because it was noticed that the baby was not feeding well. He had begun bilious vomiting, and his abdomen was distended. Upon a physical examination, his vitals were normal. Upon palpation, persistence of abdominal distension was noted, and the baby’s movements were sluggish. Bloody mucoid stools were also observed.

**Figure 1:**
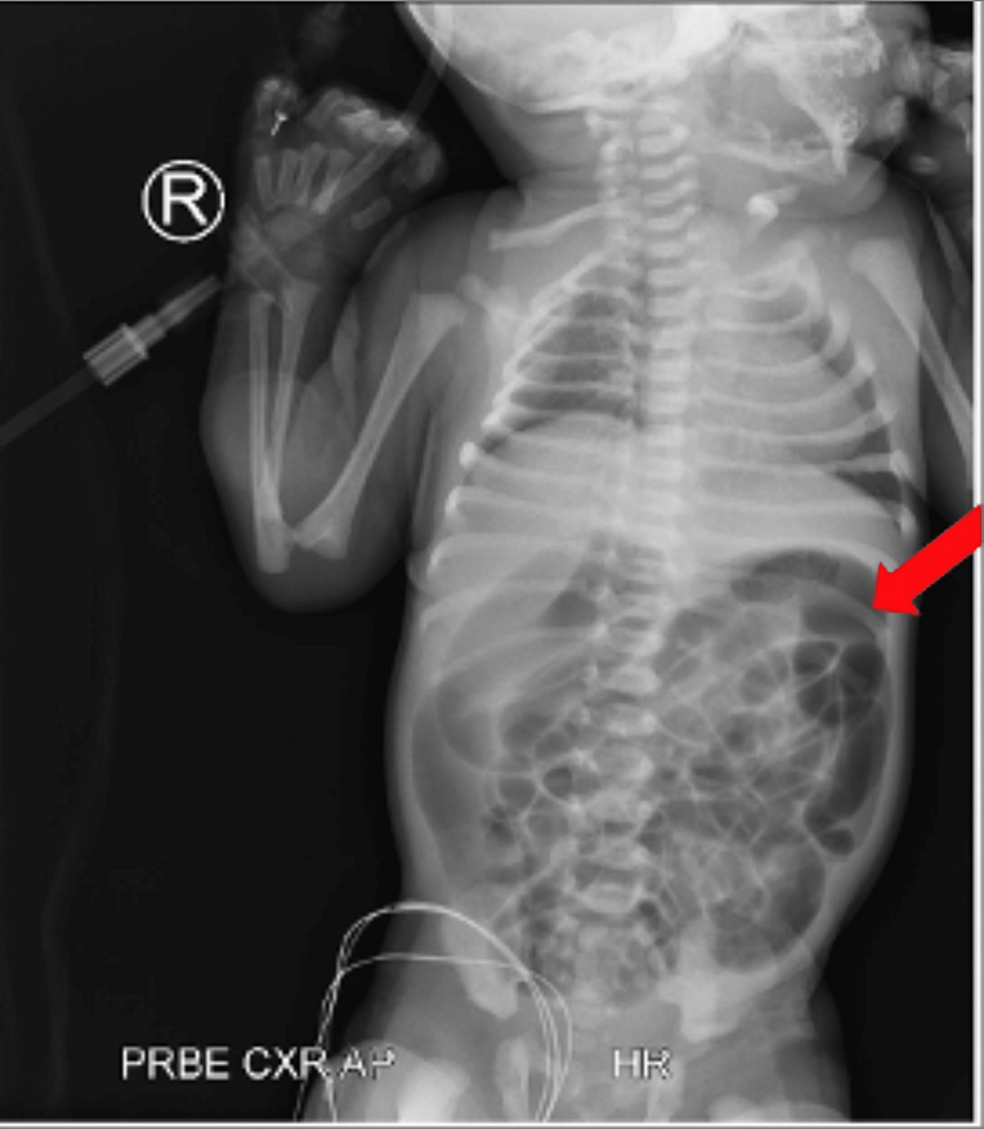
Simple abdominal x-ray of male neonate indicating diffuse air distension of bowel loops (note the red arrow)

In view of these symptoms, the baby was put under nil by mouth. A nasogastric tube was inserted, revealing a light greenish aspirate. An x-ray was done, which showed multiple loops of dilated small bowel which indicated obstruction (Figure 1). Intravenous (IV) fluids were started, and antibiotics (amoxicillin 50 mg dose every 12 hours and gentamicin 5 mg every 48 hours) were given due to the possibility of bowel necrosis and perforation.

**Figure 2:**
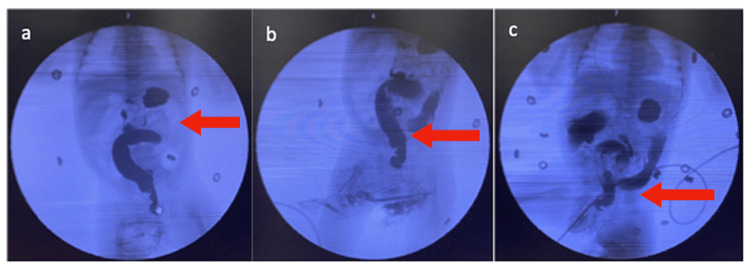
Contrast enema of baby revealing (a) distended abdomen, (b) irregular contraction, and (c) bowel obstruction

Consequently, the case was then referred to pediatric surgery. A contrast enema was performed, and Hirschsprung’s disease was suspected due to a transition in diameter between the two parts of the gut (Figure 2).

A full-thickness rectal biopsy above the pectinate line was done, and the findings were consistent with Hirschsprung’s disease, confirming the diagnosis. The reports stated that there was an absence of ganglion cells in the rectal and distal sigmoid colon, categorizing this condition as short-segment Hirschsprung’s disease. The parents were counseled about the condition, and its prognosis was outlined with the possible complications that may occur (colonic perforations and enterocolitis). The doctors, after obtaining consent, decided to perform the Duhamel procedure as the course of treatment. Prior to the surgery, an injection of vitamin K (0.5 mg) was administered to prevent any coagulation problems. The surgery went well, with 15 cm of the bowel resected.

IV antibiotics (three doses of gentamicin 5 mg every 48 hours, amoxicillin 50 mg dose every 12 hours, and cefotaxime 50 mg dose every 12 hours) along with a nalbuphine infusion (10 mg/mL) for pain relief were given for the first two days postoperatively. Oral diet was started two days postoperatively coupled with IV fluids with electrolytes, and the total length of stay in the hospital was seven days. There were no other complications following discharge, and the baby is doing well.

## Discussion

This case highlights that an early diagnosis is key to providing accurate and timely treatment for neonates with HSCR. Neonates and infants are at the greatest risk of complications compared to older children [4]. In unfortunate cases, the fluctuations in diagnostic effectiveness across all modalities can lead to failure in diagnosing HSCR and may cause children to suffer from complications later in life such as a dilated colon, severe colonic distension, and chronic constipation [1].

Abdominal distention (>90%), bilious vomiting (>85%), and failure to pass meconium within 24 hours of life (60%) are common clinical symptoms of newborns with HSCR [8]. Other clinical features include poor feeding and chronic constipation [8].

Newborns often swallow quantities of air after birth and can become distended in the large intestine in the presence of HSCR, primarily due to an obstruction at the proximal aganglionic segments [11]. Given the high suspicion of index for HSCR, imaging can aid in determining the location of the “transition zone,” a point wherein the presence of aganglionic bowel is present compared to normal colonic tissue [12]. In a study by Pratap et al. [13], the plain abdominal radiograph transition zone (PARTZ) had 96% accuracy in determining the level of transition zones when compared to standard contrast enema (84%).

Similarly, more recent studies demonstrated that barium enema had high sensitivity and specificity in detecting the transitional zone at 90% and 80%, respectively, over PARTZ [14]. Considering the efficacy of both, initially utilizing PARTZ and following up with contrast enema significantly improved the early diagnosis in our patient. However, a disadvantage to imaging techniques is the possibility of yielding nonspecific findings that do not definitively diagnose HSCR in neonates.

Despite the lack of consensus that determines the superiority of different imaging modalities, the European Reference Network for rare Inherited and Congenital digestive disorders (ERNICA) guidelines advocate rectal suction biopsies with acetylcholinesterase (AChE) immunohistochemistry as the gold standard for HSCR [15,16]. Histological results confirming HSCR include the absence of ganglion cells in the submucosal or intramuscular plexus and the presence of hypertrophic nerve trunks [17]. The findings of superiority in rectal suction biopsies are corroborated by multiple studies in the literature and are appraised as the least invasive and safest method of obtaining biopsies in neonates [16].

A retrospective study conducted by Keyzer-Dekker et al. [18] found that in 183 patients, the sensitivity and specificity of rectal suction biopsies were 83% and 97%, respectively. Similarly, rectal suction biopsies were rendered the best age-related diagnostic technique when diagnosing HSCR in neonates greater than one year of age compared to anorectal manometry in children of the same age range [19]. This finding has been supported by Croffie et al. [20] where suction rectal biopsies were shown to provide a less adequate sample to accurately identify the ganglion cells after the age of three.

Therefore, it is reasonable to conclude that the adequacy and success of rectal suction biopsy samples can be age-specific; there is usually a better yield in neonates as with age advancement, the tissue obtained would less likely contain adequate submucosa for identification of ganglion cells [20]. Yet, skepticism remains among pathologists in the popular usage of rectal suction biopsies. Though suction biopsies were deemed the safest option, given the array of complications in conventional transmural biopsies, the interpretation of results is limited to the surface of the submucosal plexus. Moreover, the accuracy of frozen section (FS) evaluations has been extensively debated. While certain studies have determined that FS should neither be the primary tool for the initial diagnosis nor the basis for performing surgery due to its reportedly high rates of incorrect diagnoses [21,22], Kataria et al. [23] found FS to accurately mark the aganglionic portions of the colon for surgery in 96.2% of rectosigmoid HSCR cases.

Fortunately, the quick diagnosis made by the consultant accounted for prompt surgical action, free of complications. The ERNICA guidelines recommend a trans anal endorectal pull-through (TERPT) or Duhamel pull-through as the definitive operation for HSCR, both of which can be performed laparoscopically [15]. In the literature, there is no comparative superiority of both diagnostic procedures in terms of complications or long-term bowel motility and a lack of data that determines the optimal timing of the pull-through surgery [24]. Therefore, the surgeon's level of expertise guides the optimal treatment for HSCR. Research also suggests that Duhamel procedures grant better visibility and may be preferred in neonatal HSCR but do not warrant superiority as TERPT confers better cosmesis [25].

Postoperative care recommends following the enhanced recovery after surgery (ERAS) protocol in optimizing care after major procedures and focuses on pain management, infection control, and nutritional supplements, which likely contributed to the rapid recovery of our patient upon follow-up [15]. Though genetic screening was not ordered, neonates with HSCR are recommended to undergo testing for trisomy 21 and other congenital malformations to monitor the risk of HSCR and potential re-operation [26]. The facilities needed to carry out these investigations may not be possible in low-resource settings, and this warrants the need to determine the cost-benefit of genetic screening in neonatal HSCR.

The favorable outcomes in our case yielded positive results in the early diagnosis and management of HSCR. This is largely attributed to the rapid time of recognition of symptoms to definitive diagnosis. However, the current literature lacks case reports of HSCR in neonatal periods. In one notable case study by Neumann et al. [27], a 17-day-old boy with a previous history of oligohydramnios was suspected of HSCR due to failure to pass meconium on the third day. The patient was hemodynamically unstable and prompted urgent treatment for enterocolitis ensued but unfortunately passed away when he relapsed after surgery.

Both case studies show that though HSCR in neonates is prevalent, it is detrimental if recognition and treatment are delayed. Prato et al. [4] corroborated the importance of early diagnosis and found that the onset of HSCR and its accompanying clinical features correlated with disease severity. As a result, it is highly recommended that close follow-up appointments and appropriate guidelines to recognize the symptoms of HSCR are necessary to ensure timely and safe recovery postoperatively.

There are a few limitations to our study. As this case report is based on a single-center experience, it lacks generalization to a larger community. While this study shows the importance of early diagnosis in HSCR and its diagnostic modalities, the sample size limits these outcomes. Moreover, genetic testing was not carried out; therefore, we could not draw conclusions about the etiology of this patient’s congenital anomaly. Further multicentric cross-section studies should be conducted to evaluate the incidence of HSCR in neonates, the efficacy of imaging and biopsy techniques, and the relevance of genetic anomalies in HSCR to yield robust data that can support the conclusions drawn from this case study.

## Conclusions

HSCR is a rare condition, which can potentially prove to be detrimental and fatal if not promptly recognized in neonatal life. HSCR is infrequent and uncommon in neonates, hence being potentially detrimental and fatal if diagnosed late. It is critical that physicians can identify the typical symptoms of the disease, which include a distended abdomen and no passage of meconium within the first 48 hours of birth. Therefore, we hope this article allows pediatricians to include HSCR as a potential diagnosis in cases of bowel obstruction, preventing any delay in the diagnosis and treatment.
